# Automated ECG Report as a Factor in the Clinical Decision Pathway for Acute Chest Pain in the Emergency Department

**DOI:** 10.7759/cureus.101785

**Published:** 2026-01-18

**Authors:** Ashok Kumar Sankaranarayanan, Firas AlNajjar, Anas Musa, Mehraj Waheeda Kuthbudeen, Afrah Ghayoor Abdul Wahab

**Affiliations:** 1 Emergency Medicine, Rashid Hospital, Dubai Health, Dubai, ARE; 2 Cardiology, Rashid Hospital, Dubai Health, Dubai, ARE; 3 Medicine and Surgery, Dubai Medical University, Dubai, ARE

**Keywords:** 12-lead automated ecg interpretation, acute chest pain, clinical decision support, electrocardiography (ecg), emergency department

## Abstract

Background

Electrocardiographic analysis algorithms have consistently evolved, becoming essential tools for physicians in diverse settings, particularly in assessing patients with acute chest pain. Moving forward, it is crucial to classify unstructured automated ECG reports into clinically relevant outcomes using advanced large language models. This approach holds significant potential to enhance an accelerated clinical decision pathway in clinical settings.

Objective

This study aims to integrate automated electrocardiogram algorithms with advanced machine learning techniques, enhancing the classification of ECG reports within emergency department settings. Specifically, it investigates how natural language processing can augment traditional methods to accelerate the electrocardiographic-directed management of acute chest pain.

Methods

Employing a retrospective observational dataset from Rashid Hospital, Dubai, spanning from June 2022 to August 2022, we analyzed 860 ECGs from patients presenting with acute chest pain. The ECGs were categorized into four classes, namely, STEMI, NSTEMI, normal ECG, and new arrhythmia using a hybrid model that combines the established Glasgow algorithm with a large language model, GPT-4. The Glasgow algorithm produced structured text inputs, which were then classified by GPT-4 using few-shot prompting (temperature = 0.2, top_p=1.0).

Results

The model demonstrates high predictive accuracy for normal ECGs, achieving an F1 score of 0.93, followed by STEMI with an F1 score of 0.80. New arrhythmias, however, present more challenges, reflected by the lowest F1 score of 0.45. Notably, the model excels in discriminating between STEMI and normal ECGs (AUC=0.92) and between STEMI and new arrhythmias (AUC=0.91). Overall accuracy was 85.9% (95% CI: 0.816-0.895)

Conclusion

The findings suggest that leveraging deep learning alongside traditional algorithms can significantly improve the rapid classification of ECGs, supporting accelerated decision-making pathways in clinical practice.

## Introduction

The computerized electrocardiogram (ECG) has made tangible strides since 1960, in the light of extraordinary efforts made by pioneers in this field [1]. The earliest evaluation of different computer interpretation programs was in 1976, which concluded that the overall accuracy of computers is 80% and should be over-read by an experienced physician [2]. Since then, an enormous transformation has happened in various computational methods in ECG interpretations. Automated ECG interpretation has markedly advanced since the early 1990s, with over 100 million ECGs interpreted annually by computerized algorithms globally as of 2006 [3]. The essential aspect of automatic and rapid identification of cardiovascular disease in all clinical settings supporting physician decisions has to be justified for this progression. However, the real challenge has been in the standardization of computer-interpreted ECG algorithms among different manufacturers, which can lead to the usage of standard terminology and maintaining the accuracy level in reporting. Recently, great efforts have been made to create a single standard for different types of ECG analysis algorithms [4]. The ECG analysis algorithms have significantly reduced the physician readers’ time and have been a formidable companion for accurate ECG interpretation [5]. Given numerous studies evaluating the role of automated ECG in acute coronary syndrome, a large experience has accumulated that permits the correlation of ischemia-induced ECG abnormalities [6-8]. It would be appropriate to take this significant progress forward if there is a clear and mutual definition of automated ECG report pertaining to the clinical outcome of interest, such as patients with acute chest pain [9]. Numerous machine learning algorithms (MLAs) have been explored for ECG analysis; however, these models often fall short in terms of explainability and interpretability. Additionally, there are significant challenges in the clinical implementation of MLAs [10,11], in contrast to rule-based algorithms that are already integrated into major electronic health record (EHR) platforms [12]. Recent transformer-based models have improved ECG signal interpretation in large datasets, but their clinical use remains limited by computational demands, data dependence, and low interpretability [13]. By comparison, our study applies a lightweight few-shot GPT-4 model that classifies free-text ECG reports in real-time emergency settings with minimal training data. Given the significant progress in automated ECG interpretation and its integration into clinical workflows, a critical next step is to address the limited adaptability of traditional algorithms when dealing with unstructured ECG outputs. Most existing models rely on waveform data and large labeled datasets, limiting their use in real-time emergency care. In this study, we aim to classify free-text ECG reports generated by rule-based algorithms into four clinically actionable categories, namely, STEMI, NSTEMI, normal ECG, and new arrhythmia, based on the established American Heart Association/American College of Cardiology (AHA/ACC) diagnostic criteria for acute chest pain. GPT-4 was selected after preliminary testing demonstrated superior performance compared to other LLMs, including fine-tuned BERT, particularly in few-shot classification of free-text ECG reports. By developing a hybrid system that combines traditional ECG outputs with GPT-4 in a few-shot learning framework, we seek to demonstrate a practical, scalable, and interpretable approach to support early triage and clinical decision-making in emergency departments. The primary aim of this study is to assess whether a few-shot GPT-4 model can classify free-text ECG reports into four predefined diagnostic categories relevant to acute chest pain. The secondary objectives include evaluating model performance metrics by class, comparing with expert consensus labels, and exploring implications for real-time emergency decision-making workflows.

## Materials and methods

Study design and sample

A retrospective observational study was conducted in the Emergency Department of Rashid Hospital, Dubai, from June 2022 to August 2022, spanning a total of 92 days. We extracted ECGs from 860 patients over 18 years old presenting with acute chest pain within 10 minutes of their arrival. Patients with traumatic chest pain and those with a known history of ischemic heart disease were excluded.

Data sources

We extracted ECG data, along with automated reports generated by the Glasgow ECG algorithm of the IMPAX heart station, from electronic health records. These data were initially captured by the Philips PageWriter TC70 machine and subsequently transferred to the IMPAX system for processing and report generation. These data were then anonymized and securely stored for analysis, ensuring compliance with Health Insurance Portability and Accountability Act (HIPAA) regulations and ethical research standards.

Data partitioning for model training and testing

**Figure 1:**
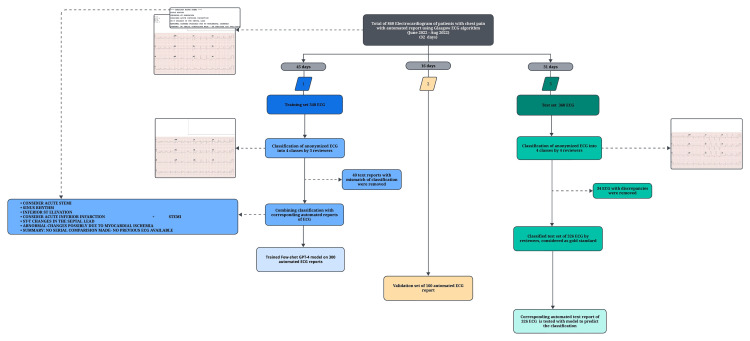
Flowchart of the study design Figure 1 summarizes the study workflow. A total of 860 electrocardiograms (ECGs) from patients presenting with acute chest pain, each with an automated Glasgow ECG algorithm report, were collected over 92 days (June–August 2022) and temporally divided into training (45 days), validation (16 days), and test (31 days) datasets. The training set included 340 ECGs, independently classified into four diagnostic categories by three reviewers. ECGs with inter-reviewer disagreement (n = 40) were excluded, and the remaining 300 automated ECG reports were used to train a few-shot GPT-4 model. The validation set comprised 160 automated ECG reports for model tuning. The test set consisted of 360 ECGs, independently classified by four reviewers. After excluding cases with classification discrepancies (n = 34), 326 ECGs served as the gold standard. Corresponding automated ECG reports were processed by the trained model, and predictions were compared with reviewer-assigned classifications.

During the 92-day study period, the 860 ECGs were sequentially collected based on time intervals: the first 45 days provided 340 ECGs for the training set, the subsequent 16 days yielded 160 ECGs for tuning model parameters and validation, and the final 31 days contributed 360 ECGs for the test dataset (Figure 1). This temporal segmentation ensured that the model was trained, validated, and tested on distinct, sequentially collected datasets.

**Figure 2:**
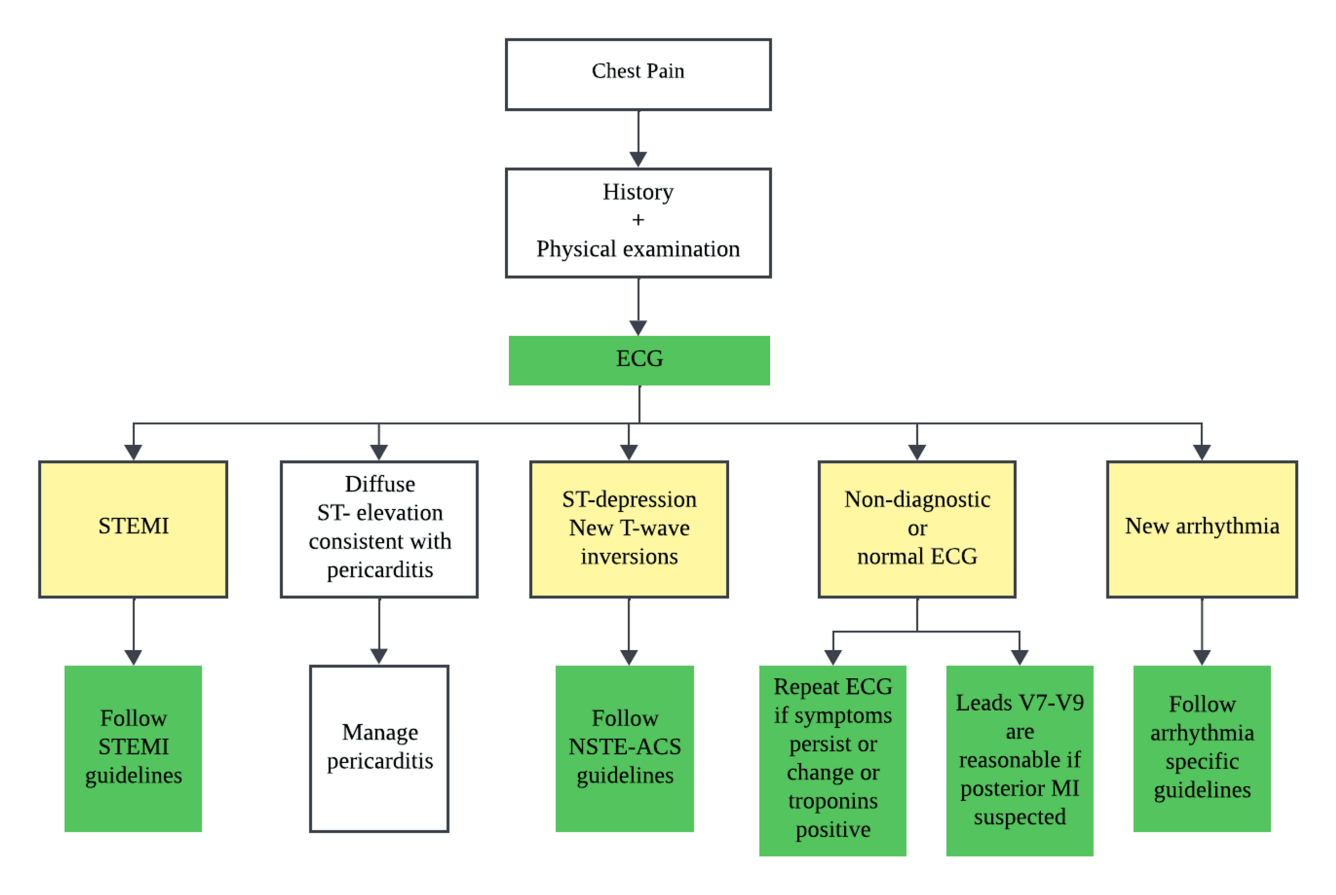
Electrocardiographically directed management of chest pain adapted from the 2021 AHA/ACC/ASE/CHEST/SAEM/SCCT/SCMR Guideline for the evaluation and diagnosis of chest pain AHA/ACC: American Heart Association/American College of Cardiology

The anonymized training set, including automated reports, was independently evaluated by two reviewers, a cardiologist and an emergency physician, both with over 10 years of clinical experience, who were blinded to each other's assessments. They classified each ECG into one of four common clinical outcomes based on electrocardiographically directed management of chest pain provided with AHA/ACC guidelines: ST-segment-elevation myocardial infarction (STEMI), non-ST-elevation myocardial infarction (NSTEMI), nondiagnostic or normal ECG, and new arrhythmia (Figure 2) [14]. A third reviewer, an emergency physician with similar experience, reconciled discrepancies between the initial categorizations of both reviewers and reintroduced the matching anonymized automated reports. This step is also instrumental in minimizing reviewer bias in the classifications. During this process, we identified a few mismatches where ECG text reports indicated ST-depression and T-wave inversion, but were classified as normal ECGs by reviewers. The 40 ECGs exhibiting these specific discrepancies were subsequently removed from the dataset to maintain the integrity of the analysis. This resulted in a cleaned final training set of 300 ECGs with an automated report and classification.

Model development and internal validation

A large language model (LLM), GPT-4, renowned for its prowess in natural language understanding and text classification, was employed to classify the ECG reports. Using a few-shot learning framework, the model was prompted with 300 annotated ECG reports and tuned using additional 160 ECGs prior to testing. The model was accessed via the OpenAI API, with the following parameters: temperature = 0.2, top_p = 1.0, max_tokens = 2048, frequency_penalty = 0.0, and presence_penalty = 0.0. These settings were selected to maximize consistency across outputs and minimize generative variability.

External validation

After training and tuning, the model was tested using a set of 360 ECGs. These ECGs were independently and blindly evaluated by four senior specialist emergency physicians, each with more than 10 years of clinical experience, and blinded to the others' assessments and the automated reports. They classified each ECG into one of four categories, with their interpretations serving as the gold standard for validation. During the review process, 34 ECGs exhibiting discrepancies among the physicians were excluded, resulting in a consolidated dataset of 326 ECGs. These ECGs, stripped of any automated reports to ensure impartial evaluation, were then used to assess the model’s capability to accurately classify automated text into the four categories.

## Results

Data distribution and sample characteristics

**Figure 3:**
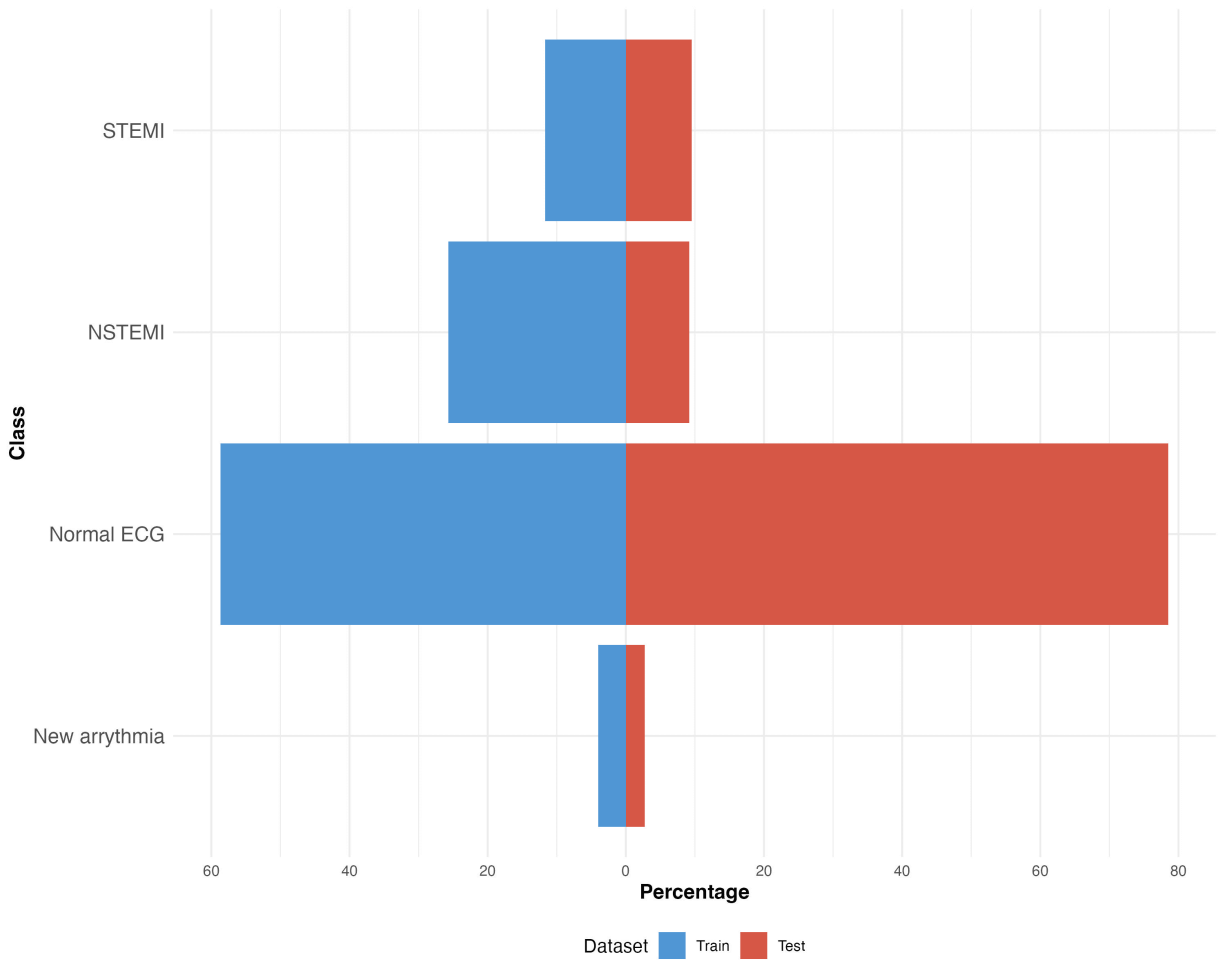
Distribution of ECG categories in the training and test sets This figure shows the percentage distribution of electrocardiogram (ECG) diagnostic classes in the training and testdatasets. Bars represent the relative proportions of normal ECG, non-ST-elevation myocardial infarction (NSTEMI), ST-segment-elevation myocardial infarction (STEMI), and new arrhythmia cases in each dataset, illustrating class balance and differences in prevalence between training and test cohorts.

Table 1 summarizes the demographic characteristics of the study population. Within the training dataset (n = 300), 58.7 of observations were labeled as normal ECGs, followed by NSTEMI at 25.7%, STEMI ECGs at 11.7%, and new arrhythmia at 4%. Conversely, the test dataset primarily consisted of normal ECGs at 78.5%, with NSTEMI ECGs and STEMI ECGs representing smaller fractions at 9.2% and 9.5%, respectively, and new arrhythmia accounting for a minimal 2.8%. These distributions are illustrated in Figure 3, which shows a predominance of normal ECG classifications and highlights the challenges in predicting less frequent conditions such as new arrhythmia.

**Table 1:** Demographic characteristics of the study population

Characteristic	Value
Number of participants, n	860
Age, mean ± SD (years)	42.9 ± 14.9
Male, n (%)	624 (72.5%)
Female, n (%)	236 (27.5%)

Inter-rater reliability analysis

For the test data, inter-rater reliability was assessed using Light's kappa and the inter-class correlation coefficient (ICC). The results revealed a kappa of 0.5, indicating fair-to-moderate agreement, and an ICC of 0.388, suggesting a moderately strong positive correlation among reviewers (95% CI: 0.333-0.445, p < 0.001). These findings confirm that the agreement among reviewers on the test data was significantly better than chance, underscoring the reliability of the evaluation process.

Model performance evaluation

**Figure 4:**
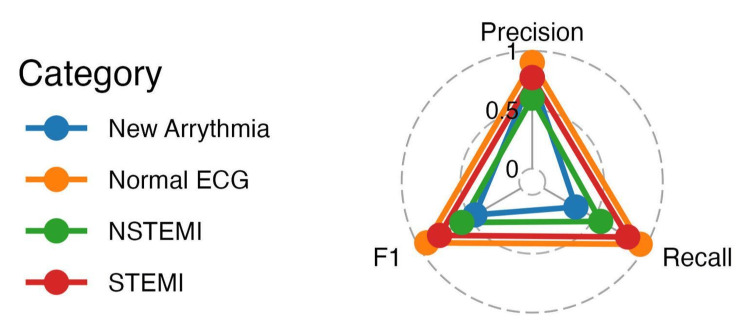
Radar plot of model performance metrics by ECG category This radar plot illustrates the class-specific performance of the trained model for normal ECG, NSTEMI, STEMI, and new arrhythmia classifications. Axes represent precision, recall, and F1 score, with values scaled from 0 to 1. Colored lines correspond to individual diagnostic categories, allowing visual comparison of performance across classes. STEMI: ST-segment-elevation myocardial infarction, NSTEMI: non-ST-elevation myocardial infarction

The model achieved an overall accuracy of 85.9% (95% CI: 0.816-0.895) in predicting the classifications of the ECGs. Despite high overall accuracy, the model struggled with new arrhythmia ECGs, which had a notably higher error rate due to their fewer occurrences. The precision, recall, and F1 scores for each category - highlighting new arrhythmia ECGs at the lowest (with 0.45) and normal ECGs at the highest (with 0.93) - underscore these outcomes. Figure 4 visually emphasizes the model’s superior performance in predicting normal ECGs compared to other classes with a radar plot. The corresponding precision, recall, and F1 values (with 95% CIs) are summarized in Table 2.

**Table 2:** Class-specific model performance metrics for ECG classification STEMI: ST-segment-elevation myocardial infarction, NSTEMI: non-ST-elevation myocardial infarction

Class	n (%)	Precision (95% CI)	Recall (95% CI)	F1 Score (95% CI)
STEMI	29 (8.9%)	0.77 (0.65–0.92)	0.83 (0.60–0.89)	0.80 (0.71–0.88)
NSTEMI	32 (9.8%)	0.60 (0.39–0.72)	0.56 (0.42–0.75)	0.58 (0.47–0.69)
Normal ECG	243 (74.5%)	0.90 (0.92–0.97)	0.95 (0.86–0.93)	0.93 (0.88–0.96)
New Arrhythmia	22 (6.7%)	0.78 (0.16–0.53)	0.32 (0.45–0.94)	0.45 (0.27–0.63)
Macro Avg	—	0.76	0.66	0.69
Overall Accuracy	—	—	—	0.859 (95% CI: 0.816–0.895)

Analysis of model discrimination capability

**Figure 5:**
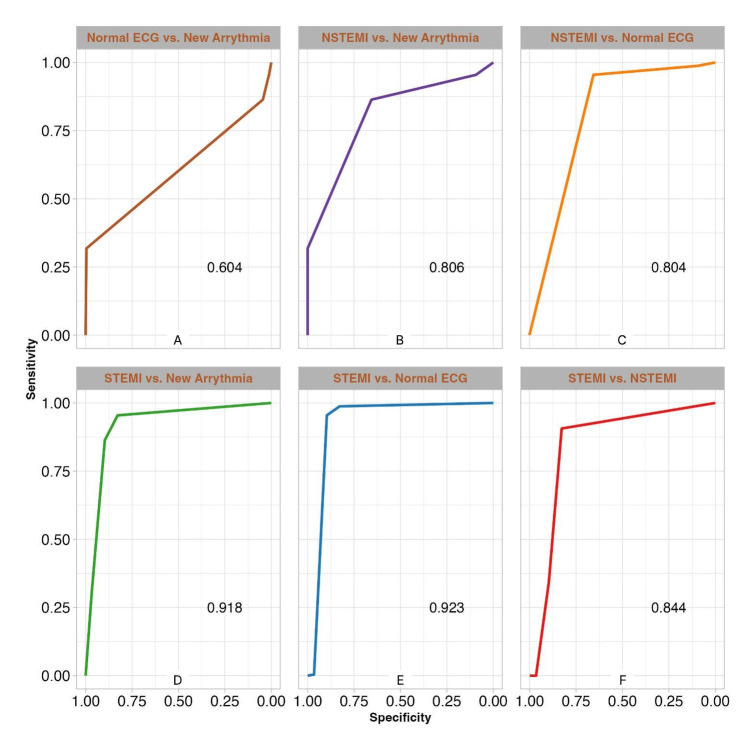
ROC curves for ECG class differentiations (A) Normal ECG vs. new arrhythmia (AUC = 0.604). (B) NSTEMI vs. new arrhythmia (AUC = 0.806). (C) NSTEMI vs. Normal ECG (AUC = 0.804). (D) STEMI vs. new arrhythmia (AUC = 0.918). (E) STEMI vs. Normal ECG (AUC = 0.923). (F) STEMI vs. NSTEMI (AUC = 0.844)

Finally, the receiver operating curve (ROC) and area under the curve (AUC) analysis further illustrate the model's discriminative power. Figure 5 displays the ROC for each category, highlighting notably more accurate predictions for STEMI ECGs compared to other categories. The model achieved its highest accuracy in differentiating between STEMI and normal ECGs (AUC=0.92, Figure 5e), as well as between STEMI and new arrhythmia ECGs (AUC=0.91, Figure 5d). However, the differentiation between normal ECGs and new arrhythmia (AUC=0.60, Figure 5a) was particularly poor, indicating specific areas where model performance could be significantly improved.

## Discussion

In this study, we evaluated the clinical utility of automated ECG reports for patients with acute chest pain by utilizing a specialized version of GPT-4. This LLM categorized the text according to established electrocardiographic protocols. With an overall accuracy of 85.89% and a kappa statistic of 0.643, the model demonstrated substantial agreement beyond chance, affirming its efficacy in ECG text classification. Pericarditis was excluded from the categories of electrocardiographic-directed management of chest pain due to its overlapping features with other conditions, which complicate accurate interpretation by computer algorithms [15]. The Glasgow ECG algorithms automated text report, which has been the focus of this study, has evolved significantly over time. It primarily employs a rule-based approach, a form of deterministic logic, which is effectively augmented by statistical analysis to enhance its diagnostic accuracy and adaptability [16]. In this research, we employed a specialized version of GPT-4, an advanced neural network model designed for few-shot learning applications, training it on a dataset of 300 examples. This LLM effectively interprets and classifies free-text ECG reports using a minimal set of input cases. Similarly, a related study employing natural language processing (NLP) techniques to classify free text from exercise treadmill tests demonstrated significant efficacy in predicting near-term cardiac outcomes [17]. This underscores the potential of advanced NLP methods, akin to our use of few-shot GPT-4, in enhancing diagnostic accuracy in cardiovascular care. A pilot study demonstrated that ChatGPT can effectively generate clinical letters with high accuracy and human-like quality from minimal input. The readability of these AI-generated letters aligns closely with those crafted by humans, indicating potential for real-world application [18]. Research has shown that integrating artificial neural networks with the Glasgow algorithm significantly enhances the sensitivity in detecting inferior myocardial infarction [19]. The model's robust performance was particularly notable in classifying normal ECGs, achieving an F1 score of 0.93. Conversely, it struggled with new arrhythmia cases due to their scarcity, which is reflected in the lowest F1 score of 0.45. This highlights the challenge of training models on less frequent conditions. Research at the University of North Carolina demonstrated that computer-interpreted "normal" ECGs, which have 99% negative predictive value, rarely necessitate changes in triage decisions. This suggests that such ECGs could minimize unnecessary interruptions for physicians [20]. Several research reports have highlighted that algorithms currently struggle to accurately identify non-sinus rhythms, including atrial fibrillation and rhythms from electronic ventricular pacemakers [21-23]. The radar plot and ROC analyses further delineated the model's strengths and weaknesses, showing excellent discriminative ability for STEMI compared to other categories, but poorer performance in distinguishing new arrhythmias from normal ECGs. This variation in diagnostic accuracy across different classes underscores the need to enhance the model's sensitivity and specificity, especially in less distinctly defined conditions. A recent study evaluated the time-consuming process of having nurses at triage screen ECGs with automated readings and then forwarding them to ED physicians for validation, underscoring the need to prioritize critical electrocardiograms [24]. Classification of ECGs, following electrocardiographic-directed management of chest pain, can enhance the accelerated decision pathway in emergency departments [25,26]. Frequent ECG reviews in emergency settings often disrupt workflow [27]; our findings suggest integrating automated classification could mitigate such interruptions, aligning with research advocating for prioritized review of critical ECGs. This approach could support regions with limited medical coverage by enhancing nurses' decision-making at the initial stage, thereby accelerating the decision-making process and improving efficiency and patient outcomes.

Future directions include expanding the dataset to further enhance the model’s robustness and precision. Implementing an ensemble approach that leverages the strengths of various LLMs could significantly improve accuracy and reliability. Training the model on ECGs correlated with patients' presenting complaints can lead to more accurate and efficient patient care. Future efforts should also focus on integrating these models into clinical workflows to validate their effectiveness in real-world settings. This includes assessing not just the accuracy but also the impact on diagnostic speed and healthcare outcomes. Employing AI-assisted ECG interpretation involves ethical considerations such as ensuring accountability, safeguarding data privacy, addressing potential biases, and maintaining transparency with patients. It is essential to tackle these factors to uphold ethical standards and patient trust in AI-enhanced diagnostics.

Limitations

This study had several limitations, primarily stemming from the fact that the model was developed based on automated reports from a single interpretation program at a single center. However, it is important to note that the University of Glasgow ECG interpretation program is widely used across various ECG machines. Additionally, the data collection occurred over a relatively short period of three months. The uneven distribution of ECG categories, particularly the scarcity of less common arrhythmias, may limit the applicability. Extending the study duration and incorporating a more diverse dataset would likely enhance the model's robustness and generalizability.

## Conclusions

This study demonstrates that a few-shot GPT-4 model can accurately classify free-text ECG reports into clinically relevant categories for patients with acute chest pain. The model performed well for common diagnoses, such as STEMI and normal ECGs, suggesting its potential as a triage support tool. Integrating LLMs with existing ECG reporting systems may improve triage efficiency and workflow support. Given that physician interpretation remains the gold standard, future work should focus on building a more balanced and representative training dataset, followed by prospective, multicenter validation, human-in-the-loop evaluation, and real-time clinical integration, to assess its role in supporting emergency decision-making.

## References

[1] Rautaharju PM (2016). Eyewitness to history: landmarks in the development of computerized electrocardiography. J Electrocardiol.

[2] Alpert JS (2012). Can you trust a computer to read your electrocardiogram?. Am J Med.

[3] Hongo RH, Goldschlager N (2006). Status of computerized electrocardiography. Cardiol Clin.

[4] Young B, Schmid JJ (2021). The new ISO/IEC standard for automated ECG interpretation. Hearts.

[5] Smulyan H (2019). The computerized ECG: friend and foe. Am J Med.

[6] Winters LJ, Dhillon RK, Pannu GK, Terrassa P, Holmes JF, Bing ML (2022). Emergent cardiac outcomes in patients with normal electrocardiograms in the emergency department. Am J Emerg Med.

[7] Cloutier JM, Hayes C, Ducas J, Allen DW (2020). Reducing delay to treatment of ST-elevation myocardial infarction with software electrocardiographic interpretation and transmission (SCINET). CJC Open.

[8] Kersten DJ, D'Angelo K, Vargas J (2021). Determining the clinical significance of computer interpreted electrocardiography conclusions. Am J Cardiovasc Dis.

[9] Faramand Z, Helman S, Ahmad A (2021). Performance and limitations of automated ECG interpretation statements in patients with suspected acute coronary syndrome. J Electrocardiol.

[10] Kao DP (2019). Intelligent artificial intelligence: present considerations and future implications of machine learning applied to electrocardiogram interpretation. Circ Cardiovasc Qual Outcomes.

[11] Mincholé A, Camps J, Lyon A, Rodríguez B (2019). Machine learning in the electrocardiogram. J Electrocardiol.

[12] Willems JL, Abreu-Lima C, Arnaud P (1991). The diagnostic performance of computer programs for the interpretation of electrocardiograms. N Engl J Med.

[13] Fu G, Zheng J, Abudayyeh I, Ani C, Rakovski C, Ehwerhemuepha L (2024). CardioGPT: an ECG interpretation generation model. IEEE Access.

[14] Gulati M, Levy PD, Mukherjee D (2021). 2021 AHA/ACC/ASE/CHEST/SAEM/SCCT/SCMR guideline for the evaluation and diagnosis of chest pain: a report of the American College of Cardiology/American Heart Association Joint Committee on clinical practice guidelines. J Am Coll Cardiol.

[15] Moak JH, Muck AE, Brady WJ (2024). ST-segment elevation myocardial infarction mimics: the differential diagnosis of nonacute coronary syndrome causes of ST-segment/T-wave abnormalities in the chest pain patient. Turk J Emerg Med.

[16] Macfarlane PW, Devine B, Clark E (2005). The University of Glasgow (Uni-G) ECG analysis program. Comput Cardiol.

[17] Zheng C, Sun BC, Wu YL (2020). Automated identification and extraction of exercise treadmill test results. J Am Heart Assoc.

[18] Ali SR, Dobbs TD, Hutchings HA, Whitaker IS (2023). Using ChatGPT to write patient clinic letters. Lancet Digit Health.

[19] Yang TF, Devine B, Macfarlane PW (1994). Use of artificial neural networks within deterministic logic for the computer ECG diagnosis of inferior myocardial infarction. J Electrocardiol.

[20] Hughes KE, Lewis SM, Katz L, Jones J (2017). Safety of computer interpretation of normal triage electrocardiograms. Acad Emerg Med.

[21] Schläpfer J, Wellens HJ (2017). Computer-interpreted electrocardiograms: benefits and limitations. J Am Coll Cardiol.

[22] Shah AP, Rubin SA (2007). Errors in the computerized electrocardiogram interpretation of cardiac rhythm. J Electrocardiol.

[23] Macfarlane PW, Mason JW, Kligfield P (2017). Debatable issues in automated ECG reporting. J Electrocardiol.

[24] Villarroel NA, Houghton CJ, Mader SC, Poronsky KE, Deutsch AL, Mader TJ (2021). A prospective analysis of time to screen protocol ECGs in adult emergency department triage patients. Am J Emerg Med.

[25] Than M, Cullen L, Reid CM (2011). A 2-h diagnostic protocol to assess patients with chest pain symptoms in the Asia-Pacific region (ASPECT): a prospective observational validation study. Lancet.

[26] Body R (2018). Acute coronary syndromes diagnosis, version 2.0: tomorrow's approach to diagnosing acute coronary syndromes?. Turk J Emerg Med.

[27] Ioannides KL, Brownstein DJ, Henreid AJ, Torbati SS, Berdahl CT (2021). Quantifying emergency physician interruptions due to electrocardiogram review. J Emerg Med.

